# Can physical activity compensate for low socioeconomic status with regard to poor self-rated health and low quality-of-life?

**DOI:** 10.1186/s12955-019-1102-4

**Published:** 2019-02-08

**Authors:** Lisbeth M. Johansson, Hans Lingfors, Marie Golsäter, Margareta Kristenson, Eleonor I. Fransson

**Affiliations:** 1 Unit for Research and Development in Primary Health Care, Futurum - Academy for Health and Care, Region Jönköping County, Jönköping, Sweden; 2 Linköping University, Linköping, Sweden; 3 The A.D.U.L.T Research Group, School of Health and Welfare, Jönköping University, Jönköping, Sweden; 4 Child Research Group, School of Health and Welfare, Jönköping University, Jönköping, Sweden; 5 Unit for Community Medicine, Department of Medicine and Health, Linköping University, Linköping, Sweden; 6 The A.D.U.L.T Research Group, Department of Natural Science and Biomedicine, School of Health and Welfare, Jönköping University, Jönköping, Sweden

**Keywords:** Physical activity, Health dialogue, Socioeconomic status, Self-rated health, Quality-of-life

## Abstract

**Background:**

Both high socioeconomic status (SES) and high physical activity (PA) are associated with better self-rated health (SRH) and higher quality-of-life (QoL).

**Aim:**

To investigate whether high levels of PA may compensate for the association between low SES and subjective health outcomes in terms of poorer SRH and lower QoL.

**Method:**

Data from a cross-sectional, population-based study (*n* = 5326) was utilized. Multiple logistic regression models were used to estimate odds ratios (OR) and 95% confidence intervals (95% CI) for the associations between indicators of SES (economic situation and educational level), SRH and QoL, as well as between the combination of SES and PA in relation to SRH and QoL.

**Result:**

Participants with high PA and economic problems had approximately the same OR for good SRH as those with low PA and without economic problems (OR 1.75 [95% CI 1.20–2.54] and 1.81 [1.25–2.63] respectively). Participants with high PA and low education had higher odds for good SRH (OR 3.34 [2.96–5.34] compared to those with low PA and high education (OR 1.46 [0.89–2.39]).Those with high PA and economic problems had an OR of 2.09 [1.42–3.08], for high QoL, while the corresponding OR for those with low PA and without economic problems was 4.38 [2.89–6.63].

**Conclusion:**

Physically active people with low SES, had the same or even better odds to report good SRH compared to those with low PA and high SES. For QoL the result was not as consistent. The findings highlight the potential for promotion of PA to reduce SES-based inequalities in SRH.

**Electronic supplementary material:**

The online version of this article (10.1186/s12955-019-1102-4) contains supplementary material, which is available to authorized users.

## Introduction

The relationship between socioeconomic status (SES) and health outcomes in terms of self-rated health (SRH) and quality-of- life (QoL) is well established all over the world [1–4]. These associations have been demonstrated for several indicators of SES for example economic situation and educational level, where both economic problems and low education have been shown to be associated with poorer SRH and lower QoL [3, 5, 6]. Notably, over the past decades SES differences in SRH have increased in several countries including Sweden [7, 8]. SRH and QoL are both subjective measures that also indicate the general state of health and wellbeing. However these measures are not equivalent nor interchangeable [9]. SRH reflects an individual’s general perception of health, including biological, social and psychological dimensions [10, 11]. QoL is a broader concept including several dimensions of life such as material resources, social functioning and social relationships, well-being, happiness, and life satisfaction [9]. Beyond the importance of a good SRH and a high QoL in and of themselves, there is also extensive evidence that SRH and QoL are potent predictors for future morbidity and survival/mortality [12–14].

Physical activity (PA) is defined as all physical activities performed by skeletal muscles that result in increased energy consumption above basal metabolic rate [15]. PA is one of the major lifestyle factors that, in addition to smoking, dietary habits and alcohol intake have a great impact on health and non-communicable diseases, for example cardiovascular disease (CVD), diabetes and cancer, as well as all-cause mortality [16–18]. In the European Region it has been estimated that lifestyle related diseases contribute to 77% of the non-communicable disease burden and 86% of all deaths [16]. High PA is also associated with a higher level of SRH and QoL [19–22]. Low PA is very common globally [23] and in Sweden a third of adults are insufficiently active according to a recent national public health survey [24]. This is especially pronounced among people with low SES, who generally report lower levels of both leisure-time PA and commuting to work PA, and also report a more sedentary behaviour [25]. Low PA could therefore be one explanation for poorer SRH and lower QoL in the groups with low SES and it is of interest to study if a higher level of PA can compensate for the observed differences in SRH and QoL related to SES [12, 26].

### Aim

The aim of this study is to investigate whether high levels of PA may compensate for the association between low SES and subjective health outcomes in terms of poorer SRH and lower QoL.

## Material and method

### Setting and data collection

This study is a part of the Living Condition Stress and Health (LSH) study which aims to prospectively evaluate causes for socioeconomic differences in health. In the present cross-sectional study, baseline data from the LSH study were used. The data were collected between 2012 and 2015 in the southeast part of Sweden. The study population includes a random sample of people aged 40, 45, 50, 55, 60, 65 and 70 years who were invited to their primary health care centre (PHCC) for a health dialogue. Those who took part in the health dialogues were also invited to participate in the LSH study. Details of the health dialogue procedure have been described elsewhere [27–29]. Compared to the standard health dialogue procedure, taking part in the LSH study included answering one additional questionnaire and providing an additional blood sample. Of the 28,702 invited people, 12,164 (42%) accepted to take part in a health dialogue and of those 6860 (56%) individuals (3880 women and 2980 men) also participated in the LSH study. The mean age was 54 years (SD of 9.9 years). Compared with data, for the general population in southeast part Sweden the LSH study population was representative regarding educational level but men aged 40 years and immigrants were slightly underrepresented.

### Analytical sample

For the present analysis, participants were excluded if they reported any of the following diagnoses (as given by a physician) as they could have a negative impact on the participants’ ability to take part in PA. The diagnoses comprised myocardial infarction or stroke, angina pectoris, chronic lung disease, rheumatoid arthritis, musculoskeletal disorders, neurological disease and depression. Out of 6860 participants, 1532 had one or more of these diseases and two participants had not answered the questions of interest for this study. After exclusion, 5326 participants remained in the analytical sample for this study.

### Indicators of socioeconomic status (SES)

In this study, economic situation and educational level were chosen as indicators of SES; both retrieved from the questionnaires. The question about perceived economic situation was formulated as “Is the economy a problem for you?” with three answer options. The responses to this question were dichotomised where the answers “partly” and “yes” were combined into “Economic problem” and the answer “no” was labelled as “No economic problem”.

Education was measured in terms of highest achieved education and responses were divided into three levels: “Elementary school” was categorized as “Low education”; “Two-year high-school/vocational school, girls’ school, secondary school or equal, three-or-four-year high-school” was categorized as “Intermediate education”; and “University degree” was categorized as “High education”.

### Physical activity (PA)

PA scores were calculated from responses to two different questionnaires about PA during leisure-time and PA commuting to work. The health dialogue performer also asked supplementary questions, which means that the measurement of PA was a combination of a questionnaire and an interview. Responses were transferred into PA “points” representing the level of PA. Further details concerning questionnaires and the calculation of PA score can be found in Additional file 1 and have been described elsewhere [27]. In this study, three levels of PA were defined, where 0–499 points denotes low PA, 500–999 points denotes intermediate PA and 1000 or more points (corresponding to at least 30 min of brisk walk per day) denotes high PA.

### Combined variables of PA and SES

Two combined variables based on PA and SES were created. The first variable was the combination of PA (low, intermediate, or high PA) and economic situation (economic problems or no economic problems). The other variable consisted of the combination of PA (low, intermediate or high PA) and education level, (low, intermediate or high education).

### Outcome variables, SRH and QoL

In the questionnaires there was one question about SRH; “How do you rate your general health status?” with five response alternatives. The answers to this question were dichotomised, where the answers “good” and “very good” were categorized as “good SRH” and the answers “fairly good”, “bad” and “very bad” were categorized as “poor SRH” [30–32].

The Cantril’s Self-Anchoring Scale “The Ladder of Life” [27], was used as an indicator of QoL. This is a global measure of QoL which has steps 0–10 where participants are asked to mark which step of the ladder of life they are standing on at the time of the data collection [33]. The top step (ten) represents the best possible life, and the bottom step (zero) represents the worst possible life [33]. In common with other studies the steps in this study between zero and ten were dichotomised, where steps zero to five were defined as “low QoL” and six to ten were defined as “high QoL” [34, 35].

### Potential confounders

Sex, age, smoking and food quality were considered as potential confounding variables since it is well known that they are associated with SES and PA, as well as with SRH and QoL. Age was divided into seven age groups: 40, 45, 50, 55, 60, 65 and 70 years old (the same as the recruitment groups).

The question about smoking habits had five response alternatives that were used in the analyses “I have never smoked”, “I stopped smoking more than 6 months ago”, “I stopped smoking less than 6 months ago”, “I smoke but not daily” and “I smoke daily”.

Food quality was captured from the questionnaire “20 questions about your food habits” that estimated the intake of hard fats, fibre and sugar. Further details concerning this measure of food quality have been described elsewhere [27]. In this study three levels of food quality were defined, where 3–5 points mean high food quality (characterised by a low intake of hard fat and a high fibre intake), 6–8 points intermediate food quality and 9–11 points depicts low food quality (characterised by a high intake of hard fat and a low fibre intake).

### Statistical analyses

Descriptive statistics were used to describe the background characteristics of the participants. Logistic regression analysis was used to estimate odds ratios (OR) with 95% confidence intervals (95% CI), to describe the associations between the indicators of SES (economic situation and educational level) and PA respectively and the outcome variables SRH or QoL. Both unadjusted estimates (Model I) and estimates adjusted for sex, age, smoking and food quality (Model II) were derived.

We also conducted stratified analyses where the association between SES indicators and the outcomes were analysed stratified by physical activity level. Interaction between SES indicators and PA was evaluated by a SES x PA interaction term in the respective model.

In the next step, the combined variables of the SES indicators and PA were analysed in logistic regression models to evaluate if a high level of PA could compensate for low SES with regards to SRH and QoL. The estimates were adjusted for sex, age, smoking and food quality. All analyses were performed using SPSS version 24 and 25 (IBM Corp, Armonk, New York, USA).

### Ethical issue**s**

The study received ethical approval from the regional ethical review board in Linköping (# 2012/336–32). All participants were properly informed about all study procedures and they gave written informed consent to participate in the study. The study is conducted in accordance with the guidelines of the Declaration of Helsinki.

## Results

### Characteristics of the study population

Characteristics of the 5326 participants included in the current analysis are summarised in Table 1, where also the indicators for PA and SES as well as the outcome variables SRH and QoL are also presented. The mean age for the participants included in the analysis was 52.9 (SD 9.7) years. The proportion of smokers was 9.2, and 21.1% were categorized in the low food quality category. The proportion of participants with high PA was 69.4%, the proportion with good SRH was 78,0%, and the proportion with high QoL was 88.3%.

**Table 1:** Background characteristics of included participant. The LSH study

Variable	Characteristics	n	Percent
Sex	Male	2404	45.1
	Female	2922	54.9
Age	40 years	1186	22.3
	45 years	419	7.9
	50 years	1275	23.9
	55 years	386	7.2
	60 years	1181	22.2
	65 years	368	6.9
	70 years	511	9.6
Smoking habits	Never smoked	3209	61.2
	Stopped > 6 months ago	1516	28.9
	Stopped < 6 months ago	36	0.7
	Smokes but not daily	148	2.8
	Daily smoker	338	6.4
Food habits	High food quality:	2174	43.9
	Intermediate food quality:	1730	35,0
	Low food quality:	1047	21.1
Economic situation	No economic problem	4363	83.2
	Economic problem	884	16.8
Educational level	Long education	1883	37.5
	Intermediate education	2470	49.1
	Short education	674	13.4
Physical Activity (PA)	High PA	3469	69.4
	Intermediate PA	845	16.9
	Low PA	682	13.7
Self-Rated-Health (SRH)	Good SRH	4071	78.3
	Poor SRH	1130	21.7
Quality-of-Life (QoL)	High QoL	4540	88.3
	Low QoL	600	11.7

### Associations between exposure variables and outcome variables

The unadjusted associations between the exposure variables economic situation, educational level or PA and the outcome variables SRH or QoL are shown in Table 2 (Model I). Among those with no economic problems or high education there were higher odds for good SRH compared to participants with economic problems or Low education (OR 3.26 [95% CI 2.79–3.81] and 1.85 [95% CI 1.51–2.28] respectively). Among those with high PA there were also, compared with those with low PA, higher odds for good SRH (OR 3.25 [95% CI 2.72–3.88). The same pattern was observed for QoL in the unadjusted analyses (Model I): there were higher odds among those with no economic problems, high education or high PA level to report high QoL compared to participants with, economic problems, low educational level or low PA (Table 2).

**Table 2:** The associations between indicators of SES and PA, and good SRH and high QoL. Unadjusted Odds Ratios (OR) with 95% confidence intervals (95% CI) in Model I and adjusted for sex, age, smoking habits, and food quality in Model II. The LSH study

	OR for good SRH				OR for high QoL			
SES	N (% good SRH)	SRH MODEL I	N (% good SRH)	SRH/MODEL II	N (% high QoL)	QoL MODEL I	N (% high QoL)	QoL/MODEL II
Economic situation	5169		4792		5111		4741	
Economic problem	867 (59)	1 ref	783 (59)	1 ref	853 (69)	1 ref	770 (69)	1 ref.
No economic problem	4302 (82)	3.26 (2.79–3.81)	4009 (82)	3.0 (2.54–3.55)	4258 (92)	5.41 (4.51–6.51)	3971 (92)	4.95 (4.06–6.03)
Education level	4930		4572		4881		4530	
Short education	659 (71)	1 ref	607 (72)	1 ref	645 (87)	1 ref	595 (87)	1 ref.
Intermediate education	2426 (78)	1.47 (1.22–1.79)	2249 (78)	1.35 (1.09–1.67)	2392 (88)	1.14 (0.88–1.47)	2220 (88)	1.1 (0.83–1.47)
Long education	1845 (82)	1.85 (1.51–2.28)	1716 (82)	1.57 (1.25–1.98)	1844 (90)	1.43 (1.09–1.88)	1715 (90)	1.27 (0.93–1.73)
Physical activity level	4894		4541		4842		4497	
Low PA	671 (60)	1 ref	628 (60)	1 ref.	664 (78)	1 ref	623 (78)	1 ref.
Intermediate PA	820 (76)	2.13 (1.71–2.66)	753 (75)	1.91 (1.51–2.42)	816 (88)	2.18 (1.65–2.90)	747 (89)	2.05 (1.52–2.77)
High PA	3403 (83)	3.25 (2.72–3.88)	3160 (83)	3.13 (2.59–3.78)	3362 (90)	2.70 (2.18–3.36)	3127 (91)	2.37 (1.88–2.99)

After adjusting for sex, age, smoking habits, and food quality, the associations between economic situation, educational level, or PA and the outcome variable SRH remained strong (Model II, Table 2). Participants who reported no economic problems or high PA had approximately three times higher odds of having good SRH compared with those who reported economic problems or low PA (OR 3.0 [95% CI 2.54–3.55] and OR 3.13[95% CI 2.59–3.78] respectively). Having a high education, compared to low education, was also associated with higher odds for good SRH (OR 1.57 [95% CI 1.25–1.98]) (Table 2). For economic situation and PA the same pattern was noted for QoL, where the odds for high QoL, after adjustment, were almost five times higher for those with no economic problems compared with those who reported economic problems (OR 4.95 [95% CI 4.06–6.03]) and more than two times higher for those with high PA compared with those with low PA (OR 2.37 [95% CI 1.88–2.99]). However, after adjustment no statistically significant association was seen between educational level and QoL (Table 2).

### Associations between socioeconomic status and outcome variables stratified by PA level

The associations between SES and the outcome variables stratified by PA levels are shown in Additional file 2. The stratified analyses showed a strong association between economic situation and both SRH and QoL in all PA strata. The associations between education and the outcomes are not as strong. When testing for statistical interaction the only statistically significant interaction between SES indicator and PA was observed for economic situation in relation to SRH (Additional file 2).

### Associations between combinations of exposure variables and outcome variables

**Fig. 1:**
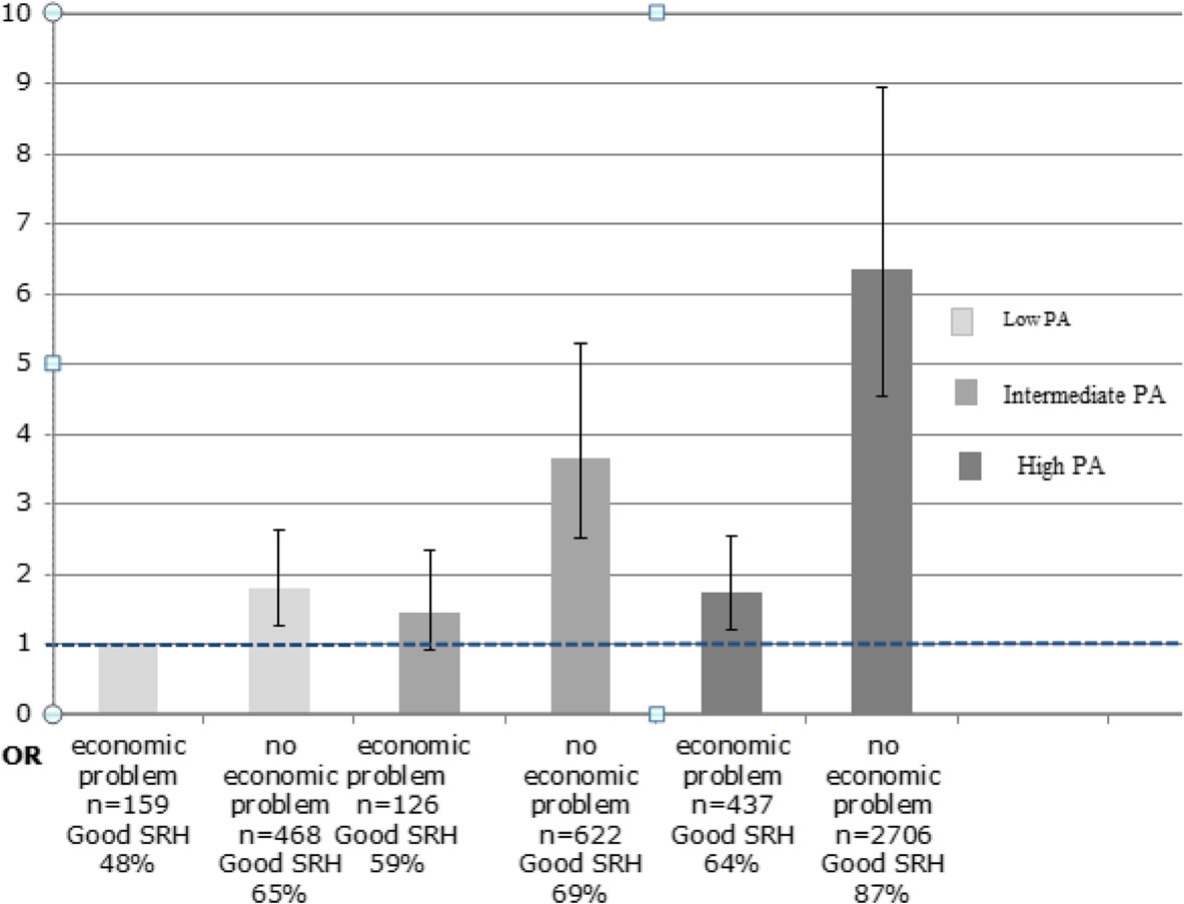
Combination of economic situation and PA, Odds ratio (OR) for good SRH. OR with 95%confidence intervals (95% CI) adjusted for sex, age, smoking habits, and food quality. The LSH study

**Fig. 2:**
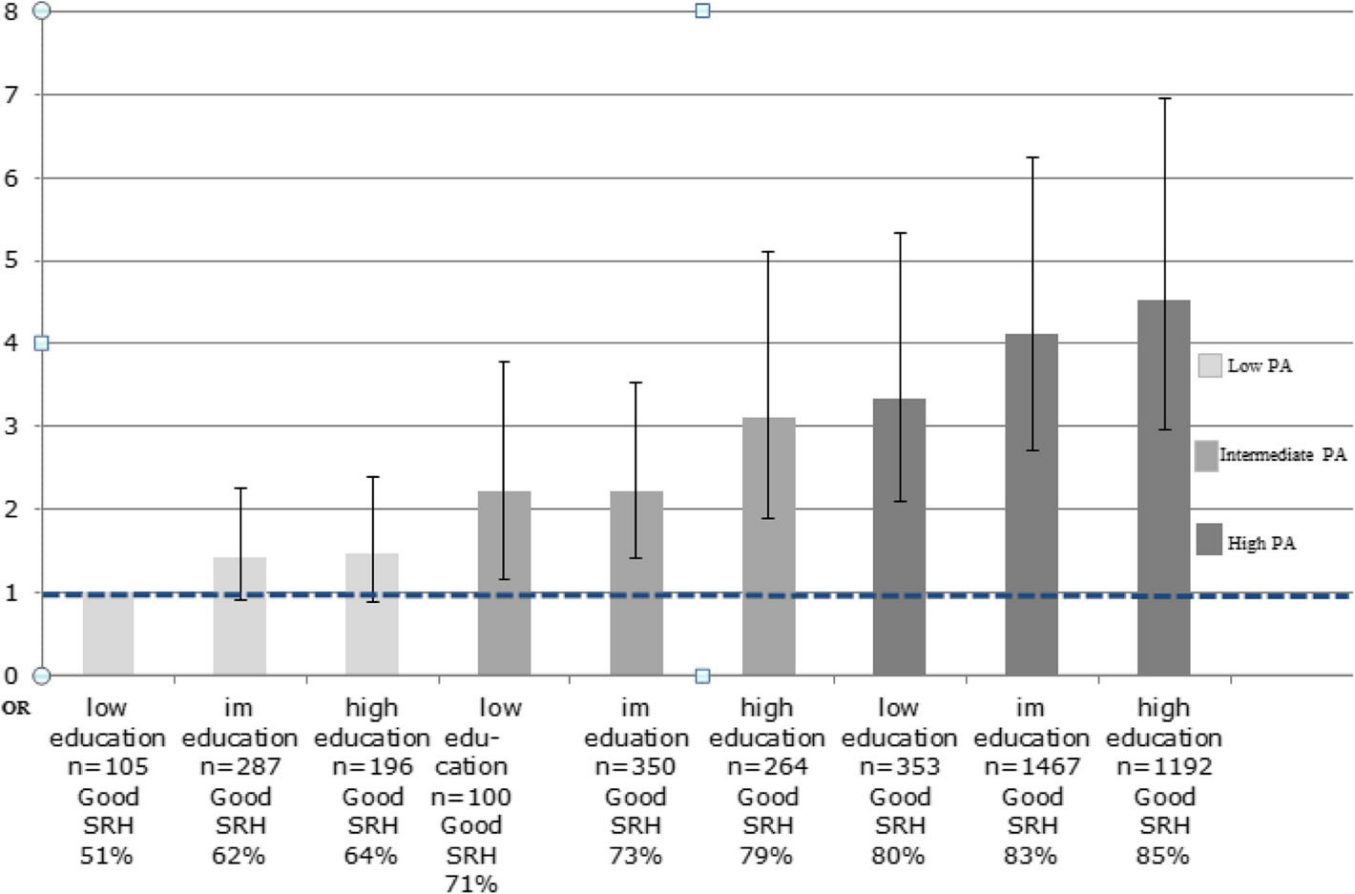
Combination of educational level and PA, Odds Ratios (OR) for good SRH. OR with 95% confidence intervals (95% CI) adjusted for sex, age, smoking habits, and food quality. The LSH study im = intermediate

**Fig. 3:**
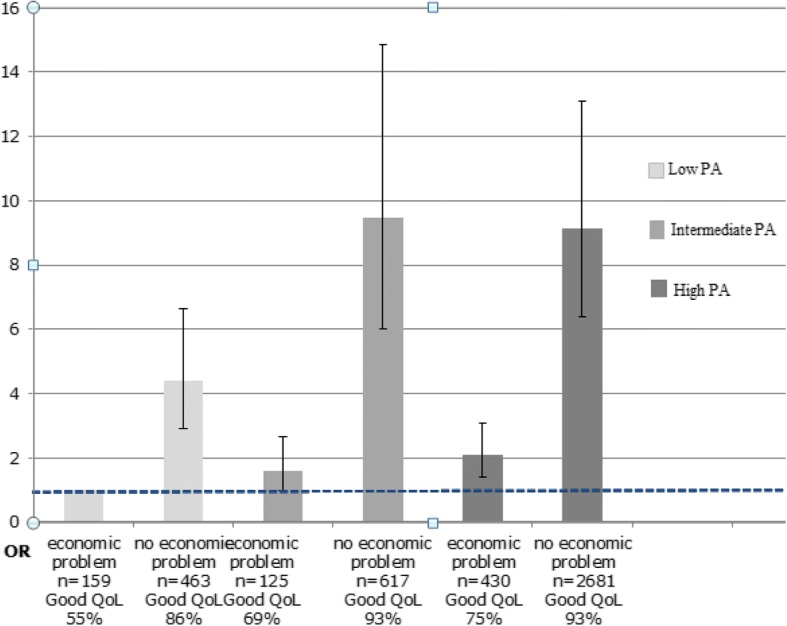
Combination of economic situation and PA, Odds Ratios (OR) for high QoL. OR with 95% confidence intervals (95% CI) adjusted for sex, age, smoking habits, and food quality. The LSH study

**Fig. 4:**
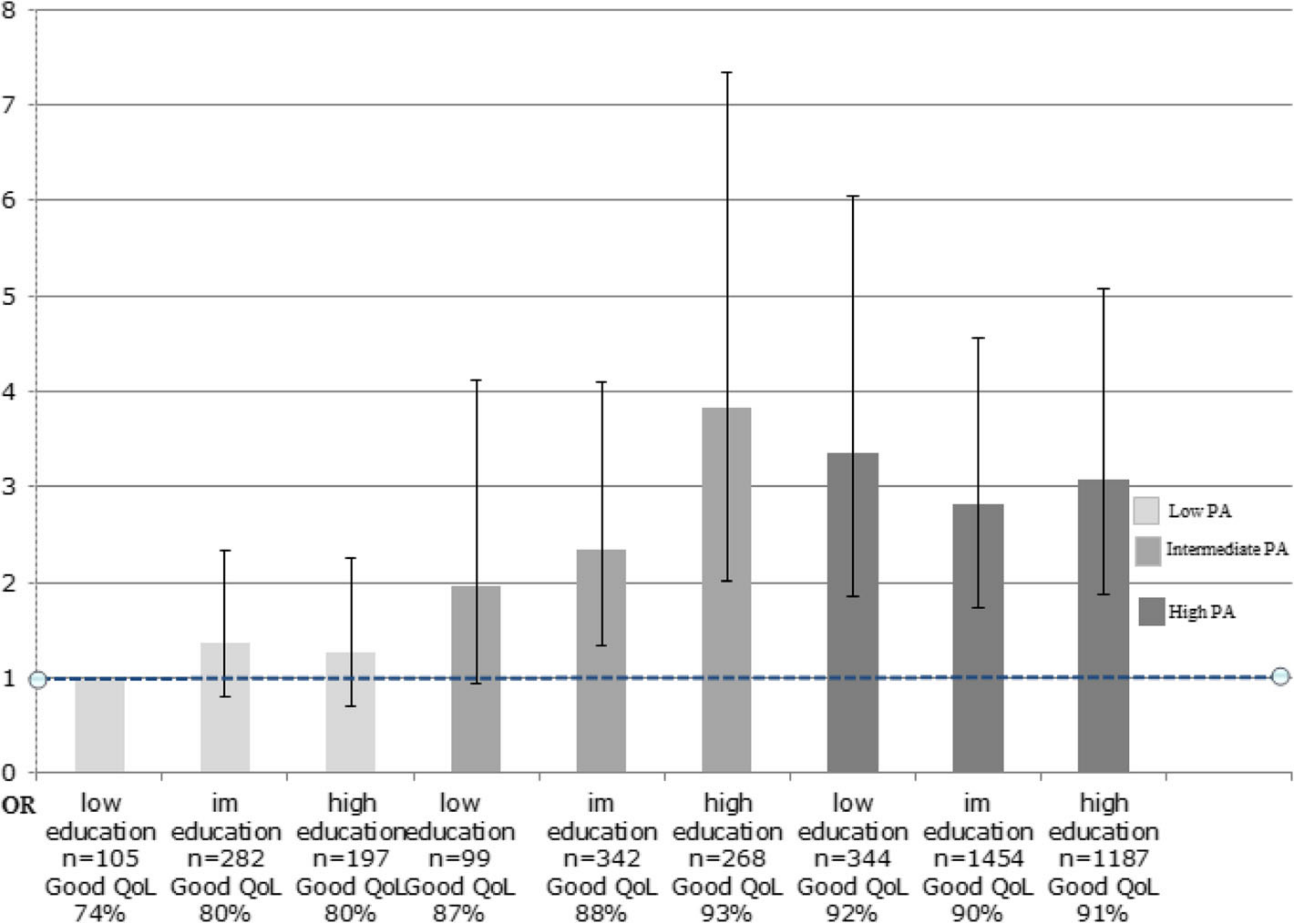
Combination of educational level and PA, Odds Ratios (OR) for high QoL. OR with 95% confidence intervals (95% CI) adjusted for sex, age, smoking habits, and food quality. The LSH study im = intermediate

The odds for the for combinations of PA and SES (economic situation or educational level) in relation to the studied outcomes, adjusted for sex, age, smoking habits, and food quality are shown in Figs. 1 and 2 (SRH) and Figs. 3 and 4 (QoL). Those with high PA and no economic problems had the highest odds to have good SRH (OR 6.36 [95% CI 4.53–8.94]) compared with the groups with low PA and economic problems. Furthermore, those with high PA who reported economic problems had approximately the same odds to have good SRH as those with low PA and without economic problems (OR 1.75 [95% CI 1.20–2.54] and OR 1.81 [95% CI 1.25–2.63], respectively) (Fig. 1).

High PA was also associated with higher odds for good SRH, in each group of educational level. Those with high PA and high education had the highest odds of having good SRH (OR 4.53 [95% CI 2.96–6.95]) (Fig. 2). Those with high PA and low education had higher odds for good SRH (OR 3.34 [95% CI 2.09–5.34] compared to those with low PA and long education (OR 1.46 [95% CI 0.89–2.39]) (Fig. 2) although the confidence intervals slightly overlapping.

The Highest odds for high QoL were observed for the groups with no economic problems and intermediate or high PA (Fig. 3), the OR for high QoL was 2.09 [95% CI 1.42–3.08], for those with high PA and economic problems compared with those with low PA and economic problems while the corresponding OR for the group with low PA and no economic problems was 4.38 [95% CI 2.89–6.63] (Fig. 3).

When combining PA with educational level the estimated ORs for high QoL are similar for those with high PA and low education (OR 3.35 [95% CI 1.86–6.04]), compared to participants with high PA and high education (OR 3.08 [95% CI 1.87–5.08]) (Fig. 4). Furthermore those with high PA and low education tend to have higher odds for reporting high QoL, as compared with the group with low PA and high education (OR 3.35 [95% CI 1.86–6.04] vs. OR 1.26 [95% CI 0.71–2.25] (Fig. 4) although the confidence intervals are overlapping.

## Discussion

In this cross-sectional population-based study we found strong associations between both SES (in terms of economic situation or educational level), and PA, with the outcomes SRH and QoL when analysed one by one. As expected booth high SES and higher levels of PA were observed for the combination of good SRH and high QoL. We also found that high PA almost offset the lower odds for good SRH, observed in groups with economic problems or with low education. However for QoL the result was not as consistent.

Our results regarding the separate associations for SES and PA in relation to SRH are in line with earlier studies that have shown that PA is related to better SRH [20] and that economic problems are associated with poorer SRH [36, 37]. However, to our knowledge, this is the first study to illustrate that PA may compensate for SES with regard to SRH and QoL in these age groups. Associations between economic problems and low QoL have also been observed previously [38]. Our results suggest that there is also a potential for individuals with low education to improve their QoL with PA.

Moreover, the odds for good SRH increased with higher level of PA in each educational level group and participants with high PA and low education reported good SRH to the same extent, as those with low PA and high education. These results are consistent with results from a study from Germany that includes older people [39].

Our results thus highlight the potential value of increasing PA to achieve good SRH both in groups with economic problems and in groups with low education. SRH is a composite measure known to be affected by both biological and social factors as well as psychological resources [10, 11]. The causes of these effects can be traced by known associations of high PA with decreased risk of several somatic diseases, but also with better mental health, improved memory functions and creative thinking [22, 40–42]. Furthermore high PA may reduce the detrimental effects of stress, improve optimism and prevent depression [43–46]. PA has been linked to salutogenesis and enhanced sense of coherence [47]. PA is also known to be related to higher QoL [42], which we also observed in this study. We found some association between economic situation and QoL but this association was not as consistent as for SRH. When combining educational level with PA the OR for high QoL, is equal or even higher for those with low education and high PA, compared with those with high education and high PA. One possible reason for slightly different results regarding SRH and QoL could be that SRH and QoL are two different aspects of the patient’s subjective experiences. QoL is a broader concept in which SRH only is one among many components.

The salutogenic theory, points out the importance of one’s own health resources and the salutogenic perspective is important in health promotion work, as a means to identify individual’s own resources to improve or maintain their health [48]. One concept that has been derived from the umbrella of the salutogenic perspective is self-efficacy [49], which means specific confidence to one’s own ability to change a specific lifestyle habit such as PA. This may be useful when supporting the efforts of individuals to achieve a higher level of PA [50, 51]. Our results provide additional evidence for the importance of supporting individuals especially those with low SES, to achieve a higher level of PA for a better SRH and higher QoL.

### Strengths and limitations

One strength of this study is that it is a well-characterised population-based study, comprising a large number of participants that are representative for the adult population as well as considering both women and men between 40 and 70 years of age. Another important strength of the study is that the included questionnaires have shown to have good validity for example for education [24], PA [52], SRH [53], for food habits [54] and smoking habits [55]. The questionnaires have also been used in several other studies prior to this one, which increases comparability between studies. We have used Cantril’s ladder of life as an indicator of QoL [33] as others have done before [56–58]. This scale has also been used to measure the closely related concept life satisfaction [59].

In the analysis we have categorised different exposure and outcome variables which were based on different scales and indices. Regarding PA the categories used in the present analysis aimed to characterize those who were mainly sedentary (Low PA) as opposed who those who are physical active to some extent (Intermediate PA) and those who reached or almost reached the WHO recommendations for PA (High PA) [17]. SRH were dichotomised, to define the group who reported good health compared to those who indicated poorer health [30–32]. For QoL the steps of the ladder of life were dichotomised, in common with other studies in this area into the groups of low QoL and high QoL [34, 35]. Furthermore economic situation was dichotomised into two groups based on self-reported information indicating problems or no problems with their economic situation. The three groups for education were defined to construct three broad categories of educational level, commonly used in Swedish context.

To reduce the risk of confounding we adjusted the analyses for sex, age, smoking habits, and diet quality Further participants who had had a myocardial infarction or stroke, angina pectoris, chronic lung disease, rheumatoid arthritis, muscle disease, neurological disease or depression were excluded, due to the possibility that the disease may have a negative impact on their ability to be physically active and may also be related to SRH and QoL. The target sample was randomly drawn from a general population. Although the response rate was low with 42% of the invited persons participating in the health dialogue and 56% of these also accepted an invitation to participate in the LSH study, the study sample is representative compared with the population in the southeast part of Sweden [60]. Also in the LSH study 72% reported good SRH compared to 73%, in a national survey [24]. In the LSH study 7% of participants were daily smokers compared to 10% percent in the national survey, 64% had high PA in LSH compared to 65% in the national survey [24]. Finally this is a cross-sectional study and we can therefore not draw conclusions about causal relationships, [61]. To further explore and confirm our findings future longitudinal or intervention studies are needed.

## Conclusions

In this population-based study of a middle-aged population we found that a high level of PA to a large degree could compensate for the negative impact from low SES in terms of economic problems and low education on SRH. For QoL the findings were not as consistent. The findings support the importance of promoting PA to reduce SES inequalities in SRH and with a potential to enhance QoL. However, as these findings are built on observational data, longitudinal or intervention studies are needed to further explore and confirm these assumptions.

## Additional files

Additional file 1: Questions about your physical activity. (DOCX 110 kb)

Additional file 2: Associations between socioeconomic status and outcome variables stratified by PA level. (DOCX 18 kb)

## Abbreviations

CVD: cardiovascular disease
LSH: Living Condition Stress and Health
OR: odds ratios
PA: physical activity
PHCC: primary health care centre
QoL: quality-of-life
SES: socioeconomic status
SRH: self-rated health
