# Supplementary figures and images for: Can physical activity compensate for low socioeconomic status with regard to poor self-rated health and low quality-of-life?
Source: Health Qual Life Outcomes. 2019 Feb 8;17:33. doi: 10.1186/s12955-019-1102-4 (PMC6368755; doi:10.1186/s12955-019-1102-4)

Appendix 1


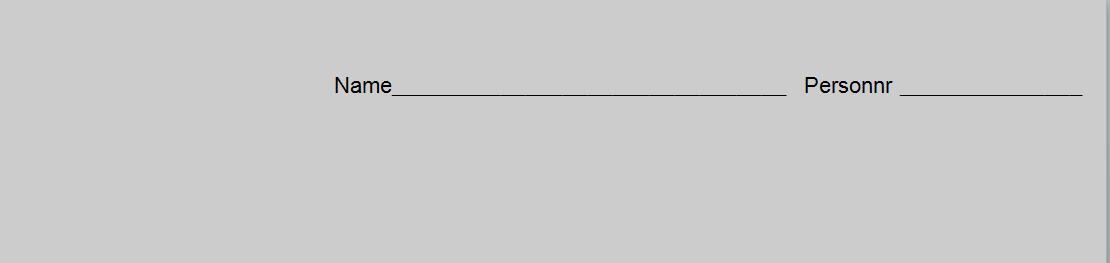


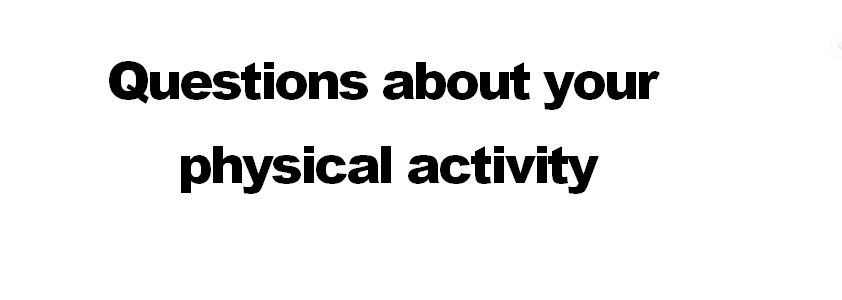


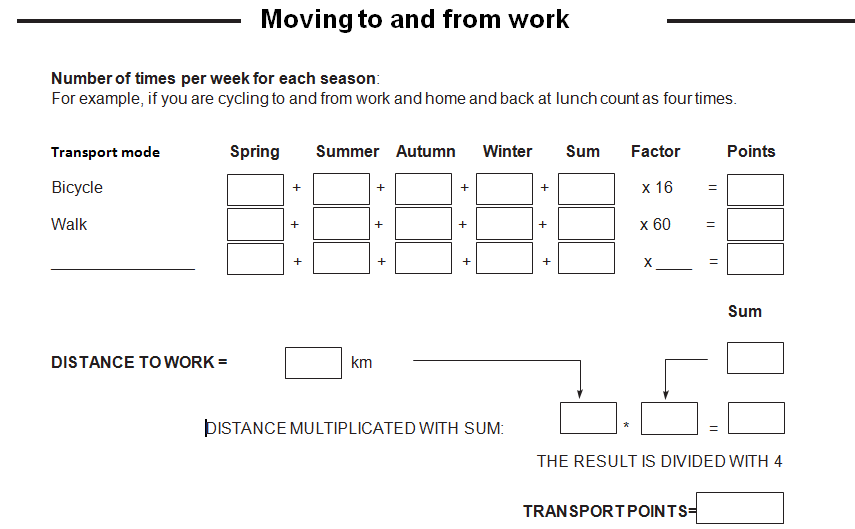


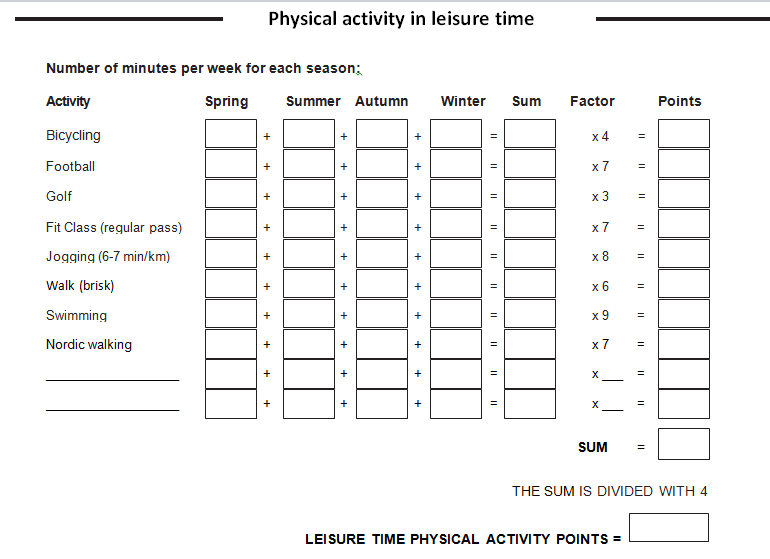


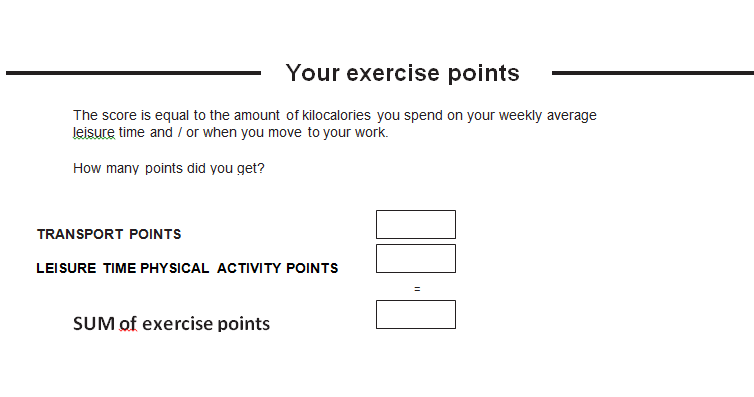

Supplement: Supplementary file 1 — Questions about your physical activity. (DOCX 110 kb) [file 12955_2019_1102_MOESM1_ESM.docx]
