# Supplementary material for: Can physical activity compensate for low socioeconomic status with regard to poor self-rated health and low quality-of-life?
Source: Health Qual Life Outcomes. 2019 Feb 8;17:33. doi: 10.1186/s12955-019-1102-4 (PMC6368755; doi:10.1186/s12955-019-1102-4)
Supplement: Supplementary file 2 — Associations between socioeconomic status and outcome variables stratified by PA level. (DOCX 18 kb) [file 12955_2019_1102_MOESM2_ESM.docx]

Appendix 2

Table 3 The associations between SES and good SRH stratified by PA levels Odds Ratios (OR) with 95% confidence intervals (95% CI) adjusted for sex, age, smoking habits, and food quality. The LSH study.

| SRH | Low PA | Intermediate PA | High PA | p-value for interaction |
| --- | --- | --- | --- | --- |
| Economic problem | 1 | 1 | 1 | 0.005 |
| No economic problem | 2.06 (1.41-3.02) | 2.59 (1.72-3.92) | 3.52 (2.79-4.43) |  |
|  |  |  |  |  |
| Low education | 1 | 1 | 1 | 0.774 |
| Intermediate education | 1.36 (0.85-2.20) | 1.08(0.64-1.82) | 1.25 (0.92-1.70) |  |
| High Education | 1.46 (0.87-2.46) | 1.56 (0.88-2.77) | 1.33 (0.97-1.84) |  |

.

Table 4 The associations between SES and high QoL stratified by PA levels Odds Ratios (OR) with 95% confidence intervals (95% CI) adjusted for sex, age, smoking habits, and food quality The LSH study.

| QoL | Low PA | Intermediate PA | High PA | p-value for interaction |
| --- | --- | --- | --- | --- |
| Economic problem | 1 | 1 | 1 | 0.556 |
| No economic problem | 4.31(2.82-6.59) | 6.04 (3.65-9.97) | 4.38 (3.34-5.74) |  |
|  |  |  |  |  |
| Low education | 1 | 1 | 1 | 0.312 |
| Intermediate education | 1.24 (0.70-2.18) | 1.10 (0.54-2.23) | 0.88 (0.57-1.37) |  |
| High Education | 1.0 (0.54-1.85) | 1.82 (0.81-4.07) | 1.0(0.63-1.58) |  |
